# Feature Tracking for High Speed AFM Imaging of Biopolymers

**DOI:** 10.3390/ijms19041044

**Published:** 2018-03-31

**Authors:** Brett Hartman, Sean B. Andersson

**Affiliations:** 1 Department of Mechanical Engineering, Boston University, Boston, MA 02215, USA; bretthartman2@gmail.com; 2 Division of Systems Engineering, Boston University, Boston, MA 02215, USA

**Keywords:** atomic force microscopy, high speed AFM, feedback-based algorithm, feature-tracking algorithm, scanning speed, frame rate

## Abstract

The scanning speed of atomic force microscopes continues to advance with some current commercial microscopes achieving on the order of one frame per second and at least one reaching 10 frames per second. Despite the success of these instruments, even higher frame rates are needed with scan ranges larger than are currently achievable. Moreover, there is a significant installed base of slower instruments that would benefit from algorithmic approaches to increasing their frame rate without requiring significant hardware modifications. In this paper, we present an experimental demonstration of high speed scanning on an existing, non-high speed instrument, through the use of a feedback-based, feature-tracking algorithm that reduces imaging time by focusing on features of interest to reduce the total imaging area. Experiments on both circular and square gratings, as well as silicon steps and DNA strands show a reduction in imaging time by a factor of 3–12 over raster scanning, depending on the parameters chosen.

## 1. Introduction

The Atomic Force Microscope (AFM) is a powerful instrument for resolving material properties and dynamics in systems at the nanometer scale [1,2,3]. While the instrument has had a large impact on a wide range of fields, its ability to image in ambient environments with a resolution far finer than the optical limit make it particularly suited for studying systems in cellular and molecular biology [4,5,6]. However, because the instrument builds an image pixel-by-pixel as it rasters across a sample, imaging rates can be quite slow, typically measured in seconds or even minutes per image.

There are many interesting dynamic processes at the nanometer scale with rates far faster than these imaging times, such as the motion of motor proteins or the dynamics of DNA-protein interactions. As a result, there is a long history of research for improving the imaging rate of AFM [7,8,9]. Developments in high-speed AFM typically follow three main approaches: improving system dynamics, using advanced controller designs and applying alternative scan paths. Since the AFM is a mechanical device, modifications to the physical components can yield substantial increases in operating speed. For example, smaller cantilevers and faster actuators have increased mechanical bandwidths [10,11,12], while advanced controllers, such as adaptive proportional-integral-derivative, feedforward compensators and multi-notch filters [13,14,15,16] permit operating bandwidths that approach or surpass the resonant peaks of the system. AFMs primarily utilize piezoelectric positioning stages that are highly resonant by nature. Therefore, consideration of the harmonic content of waveforms that drive scan trajectories can also lead to increased scan rates [17,18,19].

A complimentary class of approaches considers alternatives to the scanning process itself, seeking to reduce the number of measurements needed through either reconstruction from limited data [20,21,22] or through feedback and restriction to a limited class of samples [23].

Building upon earlier work by the authors [24,25], this paper describes and demonstrates the Local Circular Scan (LCS) algorithm. LCS is a simple feedback scheme designed for imaging edges or string-like samples such as biopolymers. It was first introduced by one of the authors as an improvement upon an earlier edge detection and tracking scheme also developed by the author, the Local Raster Scan (LRS) [24]. The basic premise behind LCS is to detect the edges of the feature of interest in real time using measurements from the instrument while driving the tip along a circular trajectory. These edge detections are then used both to center the circle on the edge and to move it along the feature. We first provide a brief review of the theory behind the algorithm and then present experimental results that illustrate an increase in imaging rate on the order of a factor of 10 using an existing, standard-speed AFM (an Agilent 5500). The key driver of the LCS scheme is the ability to detect the transition of the tip from substrate to sample. For large samples, this transition is readily apparent in either the height signal or amplitude signal of the instrument. For smaller samples, these features become increasingly difficult to detect, especially in a single circular scan where there is no larger pattern as is evident in a full image to help reveal the edges. The work described in this paper represents a significant improvement over prior results through new filtering, edge detection and image processing (described in Section 4.2), which have allowed us to apply the algorithm to samples with much smaller features, from the 20-nm gratings of the original results, down to the approximately 1.5-nm heights of Si steps and DNA. Since many other biopolymers, including actin and tubulin, are much larger than DNA, these results indicate that LCS can be used for high-speed imaging of such biological samples.

### LCS Algorithm

**Figure 1:**
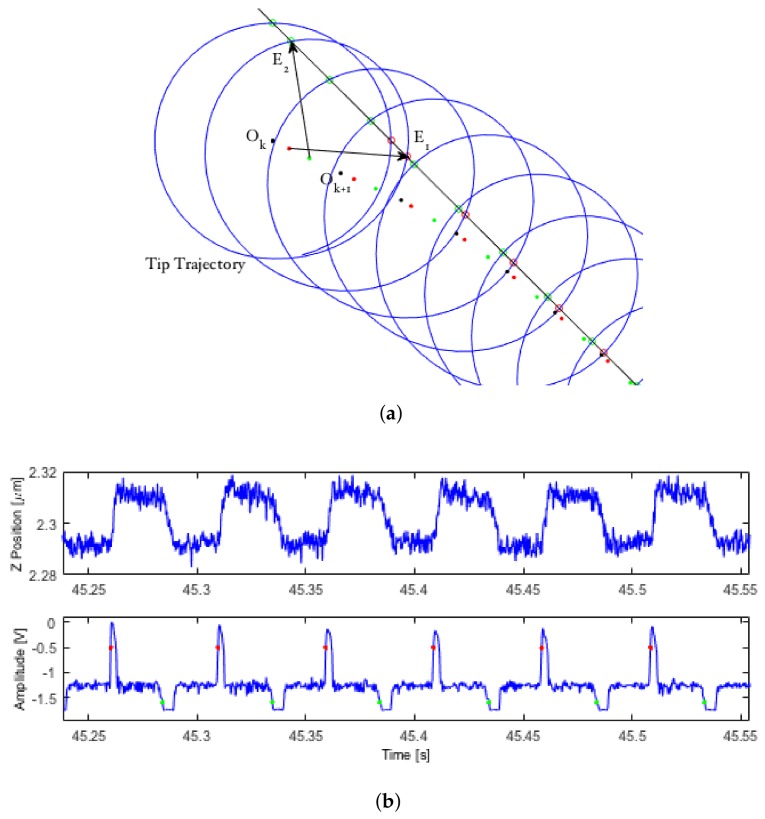
Demonstration of the LCS algorithm. (**a**) The moving reference frame at time step *k* is centered at $O_{k}$, and the positions of (detected) intersections with the sample edge are indicated with open red and green circles and define the vectors $E_{1}$ and $E_{2}$. The next center location is calculated according to Equation (1) and the reference frame smoothly moved between those points (along red and green dots) as the tip location evolves (illustration of the circle center update law); (**b**) Experimental (**top**) *z* and (**bottom**) amplitude signals from a typical scan. Red and green dots indicate detected sample edge locations in the data (*z* position and amplitude signals for a typical LCS scan along the edge of a grating).

Consider a feature whose edge defines a spatial curve, as well as a moving, local reference frame *O* attached to the center of a circle (see Figure 1a). If any part of that edge lies inside the interior of the circle, the perimeter of the circle will makes two intersections with it. Let us mark the location of these intersections with the vectors $\left(E_{1},E_{2}\right)$ and define the midpoint between these two edges as *M*, all in the local frame. These edge detection vectors can then be used to determine an updated reference frame that will allow the circle to gradually center itself on that edge while moving along the feature, according to:

(1) $$\begin{array}{c} O_{k+1}=\;O_{k}+\frac{\alpha }{2}\left(E_{1}+E_{2}\right)\pm p\frac{\left(E_{1}-E_{2}\right)}{∥(E_{1}-E_{2})∥}, \end{array}$$

where α∈(0,1) is the fraction of the distance between $O_{k}$ and *M* to move on each step, p∈(0,r) is the step distance to move along the edge of the feature and *r* is the radius of the scanning circle. In general, samples with a higher average radius of curvature require a larger value of α for reliable tracking. The choice of *p* is dependent on the desired resolution of the image, with the sole limitation that the magnitude of *p* be kept below that of *r* to avoid over-stepping the feature in non-straight regions.

In order to produce a smooth, continuous tip trajectory, the reference frame is continuously updated throughout the tip’s movement by interpolating between the starting center $O_{k}$ and the updated center $O_{k+1}$ for each point along the circle, where the total number of circle points is a function of the circle frequency ω and the command period $T_{c}$. The vector defining the path between the starting circle center and the destination circle center is given in the second and third terms of Equation (1) and is here defined separately as:

(2) $$U_{k}=\frac{\alpha }{2}\left(E_{1}+E_{2}\right)\pm p\frac{\left(E_{1}-E_{2}\right)}{∥(E_{1}-E_{2})∥}.$$

Since this update is not completed in one step, but rather is evenly distributed among each step taken to complete the circular trajectory, the interpolated circle center at each point *n* along a circle *k* can therefore be written as:

(3) $$O_{n}=O_{k}+\frac{n}{N}U_{k},\;\forall n\in \{1,\ldots ,N\},$$

where *n* is the current index number and *N* is the total number of points on each circle. This leads to a continuous trochoidal tip trajectory centered along the feature’s edge. At the completion of each circle, the starting circle center is updated to reflect the final position of the interpolated circle center according to Equation (1), and the interpolation is restarted from n=1. Figure 1a gives a demonstration of the circle center update law as LCS centers along a straight edge. Red and green circles represent positive and negative edge detections, and red and green dots represent the location of the interpolated circle center at the moment of a positive or negative detection. Figure 1b shows the *z* and amplitude signals from a typical (experimental) LCS scan during tracking of a feature’s edge.

**Figure 2:**
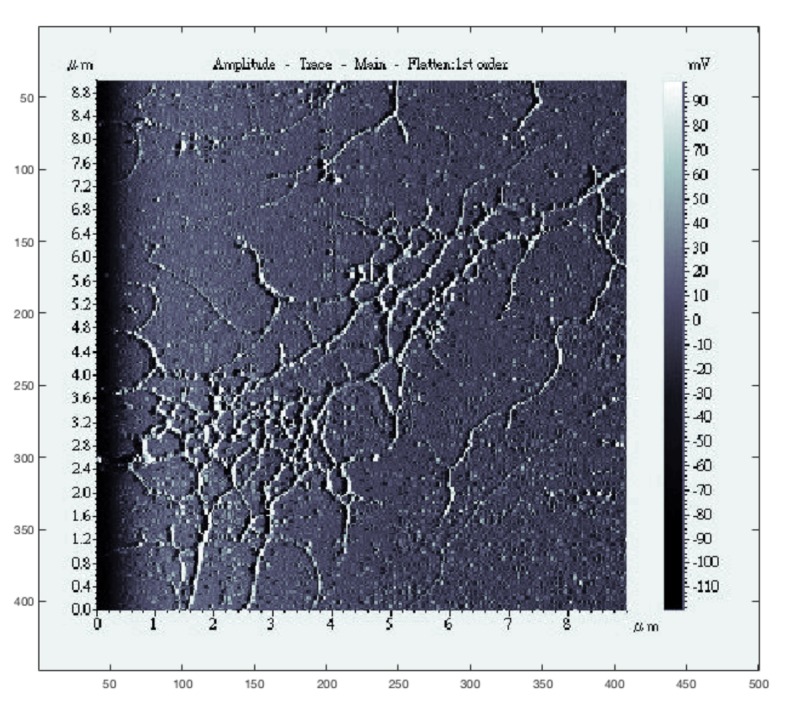
Example of an initialization image for LCS: a low quality raster image of DNA.

In practice, an LCS scan is initialized by first performing a fast, low resolution raster scan over the general region of interest, such as in the DNA scan shown in Figure 2. Once a feature of interest has been identified on the raster image, the scan can be terminated, allowing the user to click on the image at the location at which they would like the first circle to be centered for the beginning of the LCS scan.

## 2. Results

**Figure 3:**
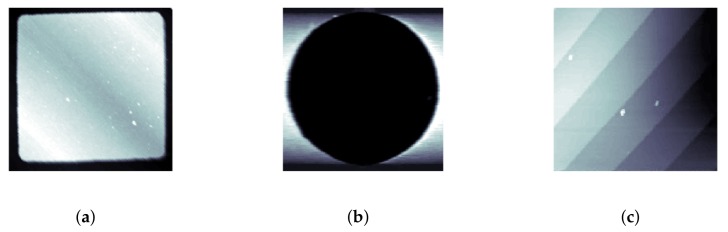
Raster scans showing the typical topology of square, circular and Si steps’ grating features. (**a**) Square grating; (**b**) Circular grating; (**c**) Si steps. (All images have been cropped from larger raster scans.)

LCS was tested on several different samples to demonstrate its ability to track features of different sizes, shapes and materials. For testing on samples with large feature sizes, a calibration grating containing both square and circular features was used (CS-20NG, Ted Pella, Inc., Redding, CA, USA). The squares were raised steps of a height of 20 ± 0.4 nm while the circles were depressions of depth 20 ± 0.4 nm; typical raster images of these features are shown in Figure 3a,b.

For a demonstration of LCS’s ability to track smaller features, as well as string-like biopolymers, experiments were performed on a silicon step calibration sample (SiC/1.5, TipsNano, Tallinn, Estonia) with a known step height of 1.5 nm, as well as on strands of λ-DNA (New England BioLabs, Ipswich, MA, USA), dried and imaged in air. The DNA preparation process is described in Section 4. A typical raster image of the silicon step calibration sample is also shown in Figure 3c.

The LCS data were preprocessed to remove standard imaging artifacts such as sample tilt and thermal drift of the instrument. In addition, the LCS scanning process does not produce data that maps to a rectangular image grid. Interpolation through inpainting algorithms was used to generate the final images from the time-series data of the LCS scan. Details of this processing are given in Section 4.3.

Table 1 provides a selection of the scanning parameters and results for these features. For all scans of the calibration grating, LCS was allowed to complete one full revolution of the feature, overlapping the path of the previous frame just enough to provide sufficient data for the removal of thermal drift effects in the final image. Details on how equivalent raster times and the corresponding Factors Of Improvement (FOI) were determined are given in Section 2.1. Specific results and images are shown in Section 2.2 and Section 2.3.

### 2.1. Methods of Comparing Imaging Times

The central premise of LCS is to scan less of a given sample than a raster scan in order to reduce imaging time by focusing solely on the region of interest. A comparison of imaging time is therefore not straightforward, and it is necessary to define some quantifiable measure of “sameness”. For these experiments, then, we consider an LCS image and a raster image to be equivalent if they were produced using the same tip speed, resolution and dimensions. The resolution of a raster image is defined by the number of scan lines used to complete the image, which is chosen to match the resolution of the LCS image. Since the spatial resolution of LCS is not uniform over the scan area, a single value is found by taking the average distance between corresponding points on two adjacent circles in the scan path, which is a function of both the scanning circle radius *r* and the step size *p*.

The dimensions of an equivalent raster scan can be defined in two distinct ways. The first assumes that the exact dimensions and orientation of the feature of interest are not known a priori. In this case, we simply define the dimensions of the raster scan as the range in *x* and *y* of the corresponding inpainted LCS image. The second definition assumes that the dimensions and orientation are precisely known and that the raster scan can be framed such that it forms a minimum bounding rectangle of the feature of interest. This second equivalence formulation should be viewed as a theoretical best case scenario for a raster scan, as it assumes exact foreknowledge of the feature of interest, as well as the ability to define the dimensions and orientation of the scan to a precise degree. In addition, LCS uses feedback to lock onto the feature, allowing repeated imaging of the same feature without drift, while the frame of a raster scan naturally drifts over time, making repeated imaging of the minimum bounding rectangle infeasible. Both definitions ignore the effects on tip speed of direction changes at the edges of the horizontal scanning range, assuming constant tip speed throughout. For the following analysis of the experimental results, both definitions and their resulting speed comparisons are considered. The Factors Of Improvement (FOI) derived from these two possible definitions will be called FOI-1 and FOI-2, respectively.

Due to the shape of the gratings, the raster framings (and therefore the factor of improvement) for FOI-1 and FOI-2 are identical. In contrast, due to the linear shape of the silicon step and DNA strand, the differences in improvement between FOI-1 and FOI-2 are significant. For the DNA strand imaged using LCS (discussed in detail in Section 2.3 and shown in Figure 8a), the “best case raster” comparison given by FOI-2 gives a factor of improvement of 3.48, while the “equal framing” comparison given by FOI-1 shows a factor of improvement of 25.42. Which comparison holds greater validity is dependent on both the hardware being used (as some systems are not able to scan arbitrarily-sized and -oriented regions) and the patience and ability of the user to perform the precise alignment assumed in FOI-2. It should be noted, however, that the tight framing in the LCS scans is inherent to the scanning process and does not depend on any prior information or user interaction.

**Figure 4:**
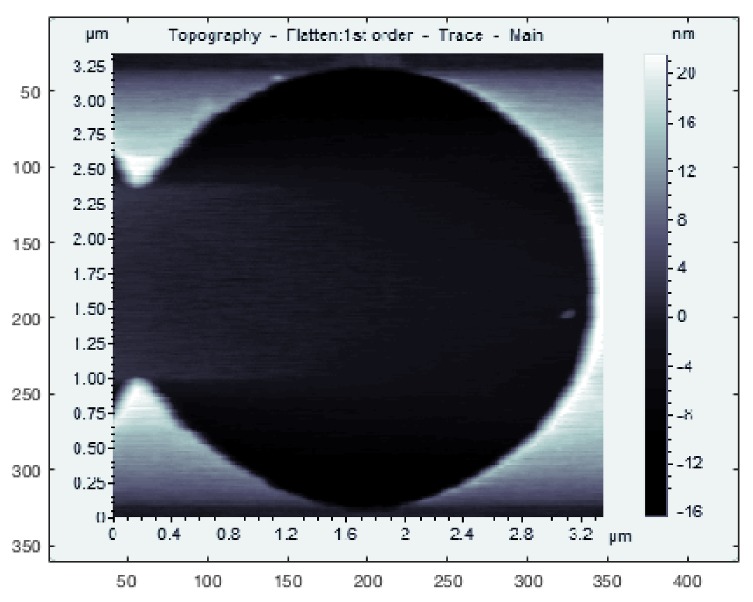
Raster image of circular grating showing the “doubling” effect due to transient dynamics in the cantilever at the sharp turnaround of the raster scan.

Additionally, even when both types of framing of a raster scan are possible and feasible for the user, a raster scan aligned so tightly to the sample will not always produce images of equal quality to an LCS scan. One of the reasons for this is the necessary abrupt changes in tip direction during a raster scan and the resulting transient effects on the image. This implies an additional advantage of LCS over traditional raster scanning, namely that it does not require the tip to rapidly change direction at any point in its scan path. The tip trajectory in LCS is continuous and smooth, regardless of the shape of the feature being tracked. This advantage becomes especially apparent when comparing LCS to raster scans for which the framing is reduced to the minimum bounding rectangle of a feature. During these scans, because the frame tightly borders the edge of the feature, when the tip changes direction from retrace to trace, the *z* actuator has not had time to fully recover from reaching the edge of the feature, leading to a streaking or cloning effect in close proximity to the edge, as can be seen in Figure 4. This effect, amplified in raster scans of small dimensions and high tip speeds, is avoided by LCS completely. Assuming the region of interest is this precise area surrounding the edge, this effect severely diminishes the utility of tightly-framed raster images. The comparisons used for FOI-2 in Table 1 are therefore not entirely reasonable. A more accurate comparison could be found by expanding the frame of the raster image until the streaking no longer infringes upon the region contained by the desired comparison frame and then cropping the resulting image to the correct dimensions. Such frame expansions increase the raster scan time, sometimes substantially if the tip speed is particularly high. We do not consider this in our comparison numbers in Table 1, and thus, in practice, the speed gains will be larger than shown in this table.

### 2.2. Results on Large Features (Square and Circular Gratings)

Experiments were performed using the CS-20NG calibration grating. The results in Table 1 show that the largest time savings, relative to a raster scan at the same tip speed and resolution, were achieved at low frequencies ω and with small radii *r*. Decreases in image resolution (that is, scans with larger *p*) lowered the absolute scanning time, but had no significant effect upon time savings relative to a raster scan. A possible explanation for the decrease in the factor of improvement seen at increased frequencies comes from the small, but non-zero delay in detecting the edge locations of the sample in the circular scan. At higher tip speeds, this delay is more significant relative to the time spent moving along a single cycle of the circular trajectory, and as a result, it pushes the location of the detection farther away from the actual edge along the circle. This leads to a circle center update step not precisely aligned with the edge of the feature which in turn causes a path length that is larger along this imagined edge than it would be along the actual edge, resulting in increased scanning time.

**Figure 5:**
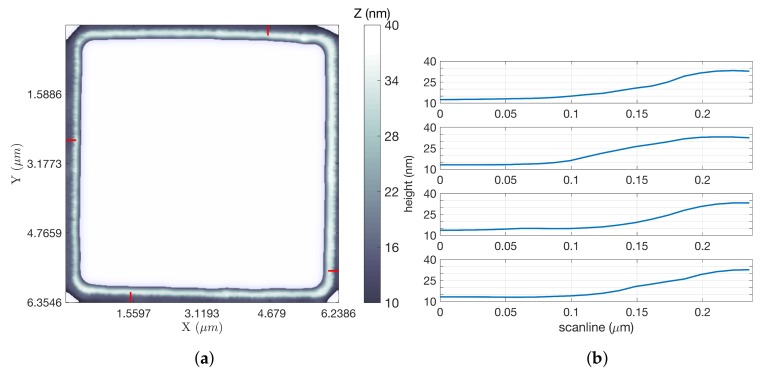
Square grating results. (**a**) LCS image. The white background indicates no information (LCS *z* image, ω = 20 Hz); (**b**) Two hundred and ten nanometer-long (17 pixel) line scans generated from along each of the four red lines shown on the LCS image, starting from the top of the rectangle and proceeding clockwise. All scans were directed outside to inside and showed step heights of (from top line scan to bottom) 20.7, 20.0, 19.6 and 19.7 nm (line scans along red lines in (**a**)).

**Figure 6:**
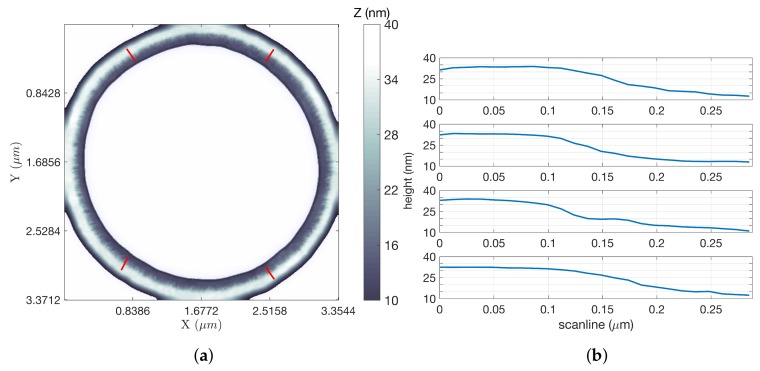
Circular grating results. (**a**) LCS image. The white background indicates no information (LCS *z* image, ω = 20 Hz); (**b**) Two hundred and ten nanometer-long (17 pixel) line scans generated from along each of the four red lines shown on the LCS image, starting from the top left and proceeding clockwise. All scans were directed outside to inside and are shown for the scans starting in the upper left and proceeding clockwise. Step heights were (from top line scan plot to bottom) 21.2, 20.4, 21.0 and 20.01 nm (line scans along red lines in (**a**)).

For scans of the gratings, the *z* signal was used to generate the LCS images. In all images shown, the white background represents no information rather than any particular height. Figure 5a shows an inpainted LCS image of a square grating, using parameters that yielded the most significant time savings over an equivalent raster scan using FOI-2 raster dimensions. This scan took 102.14 s, while a raster scan covering the same region at an equal tip speed and resolution would have taken 1193.18 s (almost 20 min), a factor of improvement of 11.68. Line scans at four locations of the square grating image (indicated by the four short red line segments in Figure 5a) were extracted from the data and are shown in Figure 5b. These illustrate a step height of approximately 20 nm. Figure 6a shows an LCS image of a circular grating using the same parameters, for which the scan took 56.31 s compared to 342.15 s for the equivalent raster, for a factor of improvement of 6.08. Line scans at four locations of this grating (also indicated by the four short red line segments) are shown in Figure 6b, again showing a step height of approximately 20 nm. For additional LCS imagery and analysis of both the square and circular calibration gratings, see [25].

In practice, the choice of the size of the scanning circle is driven by a variety of factors. For example, if the features of interest are not located on the trackable edge, but only nearby, the radius must be large enough to encompass this area. Additionally, when scanning features with very small heights, it is sometimes necessary to use a larger scanning radius to ensure that the tip speed is fast enough to produce edge detection signals that are large enough for thresholding; this is described further in the next section. With other parameters held equal, using LCS with a larger scanning radius does decrease the relative time savings over equivalent rasters; however, the difference in absolute scanning time between circles at different radii is negligible. Therefore, it is still possible to reap the benefits of LCS when large scanning areas are necessary.

### 2.3. Small Features

For the silicon and DNA samples, a derived signal referred to as ${\hat{w}}_{2}$ was used to generate LCS images. This signal is generated using an estimator based on a model of the cantilever dynamics and is described in Section 4.2.2. Note that the conversion from volts to nm was calibrated using the silicon grating scans based on their known step heights of 1.5 nm. This same calibration was then used to generate the images shown here.

**Figure 7:**
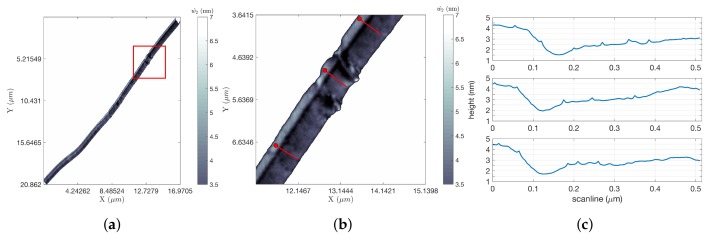
Si steps’ results. (**a**) LCS image (long scan). The white background indicates no information. The scan proceeded from the lower right to the upper left; the circular shape of the scan is revealed in the end of the image with the circular shape in the reconstructed image (LCS ${\hat{w}}_{2}$ image, ω = 100 Hz); (**b**) A zoom in of the red box in (**a**) to show the detail (zoom of the red rectangle in (**a**)); (**c**) Five hundred and fifteen nanometer-long scans generated from along the three red lines in (**b**); The top scan corresponds to the line in the top right, the middle scan to the line in the middle and the bottom scan to the line on the bottom. Each scan began at the end indicated by a circle. Step heights were approximately 1.5 nm (line scans along red lines in (**b**)).

Figure 7 shows the results of tracking a single edge of the silicon step for approximately 20 μm, after which the scan was intentionally terminated. This scan was completed in 11.50 s, yielding improvements for FOI-1 and FOI-2 of 64.95 and 4.24, respectively. The length of the scan obscures the details in the image; to highlight the resolution in the data, a zoom of the portion in the red square in Figure 7a is shown in Figure 7b. Line scans at three locations of this zoomed-in region (at the locations indicated by the three short red line segments) were extracted from the data and are shown in Figure 7c. These illustrate steps of approximately 1.5 nm. A shorter portion of this same step was completed in 3.83 s, for FOIs of 26.78 and 3.13.

**Figure 8:**
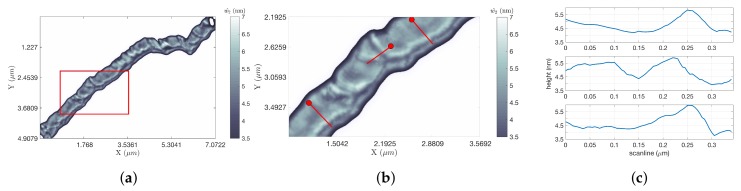
DNA results. (**a**) LCS image. The white background indicates no information. The scan proceeded from the lower right to the upper left; the circular shape of the scan is revealed in the end of the image with the circular shape in the reconstructed image (LCS ${\hat{w}}_{2}$ image, ω = 60 Hz); (**b**) A zoom in of the red box in (**a**) to show the detail (zoom of the red rectangle in (**a**)); (**c**) Three hundred forty five nanometer-long scans generated from along the three red lines in (**b**). The top scan corresponds to the line in the top right, the middle scan to the line in the middle and the bottom scan to the line on the bottom. Each scan began at the end indicated by a circle (line scans along red lines in (**b**)).

The DNA strand shown in Figure 8a took 11.88 s to scan with LCS, compared to 302.04 s or 41.39 s for the two raster framings (for factors of improvement of 25.42 and 3.48 for FOI-1 and FOI-2, respectively). A zoom of the region in the red rectangle is shown in Figure 8b, demonstrating the high degree of detail maintained in the DNA profile despite the fast tip speeds and the use of height estimation techniques. Using the calibration process described in Section 4.2.2, the measured range of heights of the λ-DNA strand shown in this figure was found to match its known range of approximately 1.5–2 nm. This is illustrated through the three extracted line scans shown in Figure 8c.

**Figure 9:**
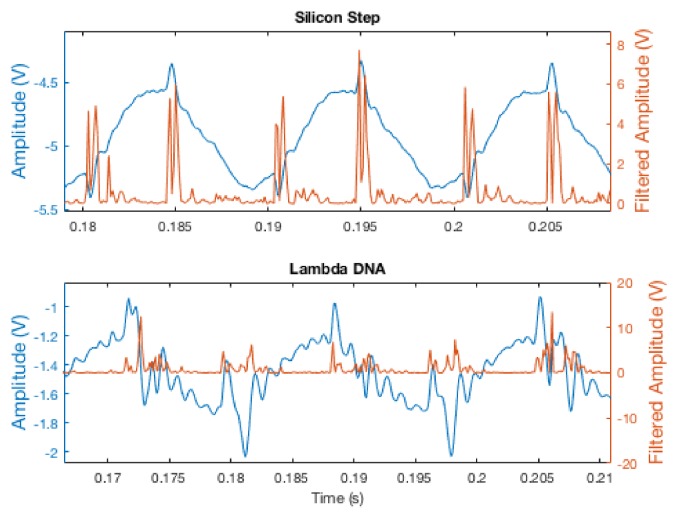
Amplitude signal during tracking of small features, pre- (sinusoidal, blue) and post-filtering (sinusoid removed, orange). Note that the sharp edges of the Si steps (**top** plot) yields sharp edges in the filtered signal, while the rounded edges of the λ-DNA (**bottom** plot) lead to a much smaller and noisier edge signal.

Despite the similar height scale of the silicon step and DNA strands used in these experiments, tracking of the silicon was considerably more reliable than that of the DNA. This is largely an effect of the relative sharpness of the edges. The edges of the silicon step, though of approximately the same height as the DNA, are sudden and distinct; there is a very clear and immediate differentiation between the lower step and the higher step. For the DNA samples, the edges often appear more gradually, reducing the effectiveness of the derivative filter in their detection (see Figure 9). Additionally, such biological samples also tend to exhibit greater variation in height along a single edge, introducing another factor that makes detection less reliable.

## 3. Discussion

While Table 1 shows that LCS can significantly reduce imaging times, it would be beneficial to know approximately how much improvement one is likely to see before committing to a scan. We have therefore formulated the estimate in Equation (4) as a rough approximation of the expected factor of improvement for an LCS scan of a given feature. Ignoring the effects of frequency and edge shape on the length of the scanning path, the factor of improvement can be approximated by:

(4) $$\mathrm{Factor}\;\mathrm{of}\;\mathrm{improvement}\approx \frac{2xyp}{bL\sqrt{{\left(2\pi r\right)}^{2}+p^{2}}}$$

where *x* and *y* are the dimensions of the raster frame needed to capture the feature, *b* is the average resolution of the LCS image and *L* is the total length of the edge of the feature.

Clearly, results can vary considerably depending on the chosen parameters, as well as the shape and orientation of the feature of interest. The scenario in which LCS is most advantageous is one in which a high resolution scan of a region in close proximity to a trackable edge is desired, particularly when the total length of the edge is small compared to the dimensions of the frame needed to capture it in a raster scan. This corresponds to small values for both *p* and *r*. However, regardless of the parameters necessary for scanning a given feature, LCS demonstrates marked improvement, provided its greatest benefit is when the dimensions and orientation of the feature are not precisely known beforehand.

### 3.1. Future Work

#### 3.1.1. Improved Edge Detection through Predictive Modeling

One of the persistent challenges with the use of LCS on small biological samples is its relative inconsistency in the detection of smooth and continuously varying edge heights. As a feature changes height over its length, the basic scheme based on thresholding of the current implementation of LCS (described in Section 4.1) is not always effective. If the threshold is set too low, false detections may dominate, while if the threshold is set too high, too many edges will be missed. In either case, tracking may be poor or lost altogether. A possible solution for this issue is to develop a scheme to dynamically alter the threshold during the course of the scan based on predictions of its shape from past measurements. Additionally, if this scheme were to take into account the sinusoidal shape of the detection signal discussed in Section 4.2.1, it would prevent the need for the filtering currently performed, leaving a more accurate and reliable detection signal.

#### 3.1.2. Multi-Frame Imaging

A promising application of the LCS algorithm is in multi-frame AFM imaging. Because the scanning region is defined through feedback during the scan itself, rather than by predefined spatial boundaries, it is possible with LCS to continually scan a feature without concern for drift shifting the feature out of view over time. By scanning back and forth indefinitely over the same feature using the feedback inherent in LCS, it is possible to create frame-by-frame movies of the evolution of a feature over time, even if it were to move slightly throughout the sample, as may be the case with active biological samples. This may be particularly powerful when studying samples such as molecular motors, polymerases or similar objects that move along biopolymers. One exciting possibility enabled by the detection-based scheme underlying LCS is the ability to follow and probe the dynamics of such objects moving on biopolymers at speeds where the resulting images are of low quality, but the resolution of the feature edges (and thus the location of the dynamic object over time) remain well resolved. A simple modification of LCS would allow for the automatic centering of the frame on this dynamic feature, allowing for high-speed and long-duration movies of the motion.

## 4. Materials and Methods

Data acquisition and implementation of the LCS algorithm was performed using a compact Reconfigurable Input-Output (cRIO) system (cRIO-9082, National Instruments, Austin, TX, USA), with software written in LabVIEW (National Instruments, Austin, TX, USA),interfaced to an existing AFM (Agilent 5500, Santa Clara, CA, USA). The LCS implementation controlled the lateral directions of the microscope. Imaging was performed in intermittent contact (tapping) mode using the manufacturer-provided controller. Cantilevers were loaded into the holder of the AFM, and the tapping mode controller was tuned to that cantilever’s specific characteristics using the built-in auto-tune software of the instrument. Image processing was performed offline using MATLAB (MathWorks, Natick, MA, USA).

Samples used for these experiments included a calibration grating (CS-20NG, Ted-Pella, Inc., Redding, CA, USA), a silicon step calibration sample (SiC/1.5, TipsNano, Tallinn, Estonia) and λ-DNA (New England BioLabs, Ipswich, MA, USA). For preparation of DNA samples, the following procedure was used: λ-DNA was diluted in purified water to a concentration of 10 μg/mL, deposited on a freshly-cleaved mica substrate (9.9 mm, PELCO Mica Discs, Ted-Pella, Inc., Redding, CA, USA) in 15-μL quantities, incubated for 5 min, then rinsed with 1 mL of purified water and allowed to air dry for 48 h before imaging in air.

### 4.1. Edge Detection Methods

The choice of an edge detection signal is vital for a proper implementation of the LCS algorithm. To fulfill this need, we make use of the amplitude signal of the AFM’s tapping mode. This signal represents the error between the amplitude set point and the measured value. When the tip encounters a positive edge, such as when moving up from substrate to sample, the amplitude of the cantilever’s oscillations suddenly and momentarily decreases before the controller is able to compensate for the change in elevation and drive it back to the setpoint, leading to a sharp spike in the error. Likewise, at a negative edge, the tip moves down from sample to substrate, causing a sharp increase in amplitude and another (now negative-going) spike in the error. It is important to note that the speed of the tip plays an important role in this detection. If the tip is moved slowly relative to the bandwidth of the controller, the amplitude signal remains at its setpoint during the entire scan and there are no spikes at the sample edges. Similarly, because the amplitude signal must be demodulated from the sinusoidal drive signal when tapping mode imaging is used, if the speeds exceed the bandwidth of the demodulator, then the signal once again becomes flat. A detailed discussion of the effects of scanning speed on both the height and amplitude signals of the AFM can be found in [26].

#### 4.1.1. Thresholding

By placing upper and lower thresholds on the amplitude error signal, the location of the edges of the sample and whether each detection represents a positive or negative edge relative to the tip’s path can be determined from these temporary spikes (see Figure 1b). These threshold values are user-defined and can be initialized based on prior knowledge of the sample and further refined mid-scan. Whenever an edge is detected, the location of the detection is stored. If a pair of edges is detected along a single circle, these locations are used to calculate the circle center update step by Equation (1), where a positive edge is $E_{1}$ and a negative edge is $E_{2}$. As discussed in Section 4.2.3, this positive-negative edge distinction is not applicable to smaller string-like samples and additional predictive steps are needed to ensure the tip maintains a consistent directionality along the sample edge.

If only a single edge (or no edge) is detected, the circle center will not be updated and the tip will continue to trace a path around the current circle center. This indicates that the circle has either lost track of the edge, has reached the end of the feature or a change in the topography of the feature requires an adjustment to the detection thresholds.

#### 4.1.2. Basic Filtering

Although the calibration gratings used for the experiments in Section 2.2 have relatively sharp edges, this is not always the case, especially with biological samples such as the DNA strands seen in Section 2.3. To avoid redundant detections due to rough or uneven edges, a filter is implemented to ensure that after the detection of a positive edge, subsequent positive edge detections are ignored until a corresponding negative edge is detected, with the same structure in place following a negative edge detection. Additionally, following any detection, processing of edge detections is momentarily paused to prevent false detections resulting from possible fluctuations in the amplitude signal after encountering an edge.

Clearly, a clean sample leads to clean detections and reliable tracking. However, it is unreasonable to expect that a sample can be made entirely free of unwanted debris, and a tracking algorithm such as LCS must be robust enough to counter the possible negative effects from such particles. Encountering dust and debris on the sample presents the possibility of false detections, which may lead to a wobbly scanning path, loss of tracking or stalling. To reduce the risk of such false detections, an optional, user-defined parameter can be applied to specify the region of the circle on which a positive or negative edge detection will be considered valid, defined by the angle θ measured relative to the circle index number *n* at which the detection occurred on the previous circle. However, use of this parameter is not always advantageous as it puts a limit on the maximum angle of edge curvature that LCS is able to follow. Thus this feature should be switched off for samples in which sharp corners and sudden changes in heading are expected.

### 4.2. Extension to Smaller Features

For smaller features, with edge heights in the single nanometer range, LCS requires modifications to both the detection scheme and the post-processing/image generation procedure. These changes are a result of the height and shape of the features, as well as the scan speeds required to obtain strong detection signals.

#### 4.2.1. Effects of Sample Tilt

On samples with smaller features, the effects of sample tilt on the amplitude and height signals within a single circle are large relative to those of the target edge. As the tip moves along its circular trajectory, the local sample tilt is projected into the amplitude signal as a sinusoid at the frequency of the circle scan. When the height of the target edges is large (relative to the sample tilt across the radius of the circle), the relative magnitude of this sinusoid is inconsequential. However, for smaller edges, the detection signals become buried in this sinusoidal wave, rendering the simple thresholding approach used for large features ineffective.

There are several approaches that could be taken to resolve this issue, the most direct of which is to filter out the sinusoid, leaving the sharp detection signals in a form that can be used for thresholding. A number of methods for implementing this signal correction were considered and tested, including a notch filter, a finite impulse response (FIR) filter and a phase-locked loop. However, these approaches, while removing the sinusoid, each had their own side effects that caused additional detection issues.

Ultimately, a simple derivative filter was implemented, which allowed detections to be processed at the same speed as the original amplitude signal while maintaining relatively sharp edges for detection. The amplitude signal was run through this derivative filter, then squared to obtain sharper peaks, as well as to ensure a consistent “positive-edge-only” thresholding scheme. Figure 9 shows the amplitude signal during tracking of both a silicon step and a strand of λ-DNA, before and after filtering. For small but sharp edges, as in the Si-steps sample, this scheme leads to distinct signature from the edges. When the transition from surface to sample is less sharp, as with the λ-DNA, the edge signature is present, but significantly less pronounced. To provide a more accurate representation of the amplitude signal for image generation, an FIR filter was used in post-processing to remove the sinusoidal component of the original amplitude signal.

#### 4.2.2. Sample Profile Estimation

Even after filtering, small sample edges produce small detection signals, which are difficult to threshold once they near the scale of sensor noise. The magnitude of these signals can be increased by increasing the tip speed (which leads to a larger change in the amplitude signal), and this has the additional unwanted effect of reducing the accuracy of the height signal as the tip speed approaches the bandwidth of the *z*-piezo controller. At such high speeds, it becomes necessary to consider the information we hope to gather from the scan, namely whether we require a fully-calibrated estimate of the absolute sample height, or whether an uncalibrated, relative-scale representation of the sample is sufficient.

For the case of the relative scale, it is often acceptable to generate an image using the AFM’s amplitude signal, from which we can generate a reasonable approximation of the sample profile itself, provided that the tip is moving at a high enough speed that it will not immediately recover to its setpoint after impacting an edge. For a calibrated, absolute-scale image, it is necessary to use more advanced estimation methods to generate a valid representation of the sample height. For this paper, we make use of a sample profile estimator developed by one of the authors. Referred to as the ${\hat{w}}_{2}$ signal, the estimator is briefly described in the Appendix A. Details can be found in [27]. Calibration of the ${\hat{w}}_{2}$ estimate can be obtained by scanning a feature of known height and finding the slope and intercept of the best fit line between two points of known relative heights. For this paper, a silicon step with a known height of 1.5 nm, as shown in Figure 7a, was used for this calibration.

#### 4.2.3. Edge Detection of String-Like Samples

When tracking the edges of large features, the tracking direction can be easily and consistently maintained by setting the trajectory to move in the direction of either the positive edges or the negative edges. For string-like biopolymers such as DNA, this is not possible, since the scanning circle is large relative to the feature, resulting in a pair of positive and negative edges on each extreme end of the circle, rather than a single distinct positive or negative edge. Furthermore, the negative edges follow closely behind the positive edges and are less distinct. As a result, they become lost in the amplitude signal at higher scanning speeds. It is therefore necessary to use a predictive approach to maintain a consistent tracking direction.

This is accomplished by marking the first edge detection of the scan as $E_{1}$ and the second as $E_{2}$ and maintaining a trajectory that follows the smoothest path by assigning the $E_{1}$ tag in any given circle to the detection that is nearest (in the sense of its circle index) to the $E_{1}$ edge from the previous circle, making the same relative assignment for the $E_{2}$ edge. This serves a dual purpose of maintaining a consistent scanning direction without the need for a positive-negative edge distinction, while also reducing the chance of tracking loss due to sample debris.

### 4.3. Image Processing

Non-raster methods such as LCS require a different approach to image processing than raster scans to account for and correct irregularities and artifacts in the collected data and to produce a standard rectangular image. Previous work with images produced by LCS has demonstrated that the most common issues, namely sample tilt, thermal drift and unevenly-spaced data, can be reliably corrected with straightforward post-processing techniques.

#### 4.3.1. Tilt Correction

Effects of sample tilt are easily corrected in raster images by subtracting the best fit line from each scan line or the best fit plane from the complete image. In LCS, there are no regularly-spaced scan lines. It is therefore necessary to fit a plane to the entire dataset to account for tilt in the sample. For large features, this amount of correction is sufficient. For images of smaller features, for which the sample tilt local to each individual scanning circle is significant (see Section 4.2.1), it is also necessary to apply additional filtering to the height/amplitude signal to remove its sinusoidal component (described above in Section 4.2.1).

#### 4.3.2. Thermal Drift

Thermal drift due to temperature differences among the different components of the AFM can cause additional skewing of the height data. In LCS images, this can be corrected by taking advantage of self-intersecting scan lines. If multiple frames are scanned in the same region, the points of intersection of the scan lines between each frame, as well as those between adjacent circles in the scan path, can be identified, and the change in height over time at those locations can be calculated. The best fit line (in a mean-square sense) to that height change can then be found and subtracted from the scan. It has been found that correcting for thermal drift prior to performing tilt correction yields better results.

#### 4.3.3. Selective Sampling and Interpolation

In raster scans, images are typically generated using data from only either the trace or retrace portion of the scan lines in order to avoid inconsistencies due to differences in tip-sample interactions on either side of the tip. We are able to perform this same differentiation with LCS by utilizing only the data obtained near a transition from substrate up to sample and discarding that obtained in the vicinity of a negative edge. This is achieved by dividing each circle in the tip’s trajectory into two halves based on the location of the positive edge detections. The half of each circle centered around a positive edge detection can be called the “trace”, while the remaining half can be called the “retrace”. By creating an image solely from the trace circle segments, we are able to achieve a similar effect to focusing on trace or retrace data in a raster scan, while maintaining the same resolution defined by the step size *p*.

Perhaps the largest consequence of a non-raster scanning pattern is the unevenly-spaced data, which require interpolation to develop a complete image. There are numerous image reconstruction methods available for use with data obtained from non-raster scanning patterns, and the ideal choice largely depends on the sample under study. A comparison of various methods, including inpainting and compressive-sensing-based reconstructions for AFM images can be found in [28,29]. For all images in this paper, we made use of heat equation inpainting to carry out interpolation, as it produced reliable and high quality images at a low computational cost [30].

## 5. Conclusions

In this paper, we have demonstrated the use of LCS as a viable technique for high-speed AFM and its effectiveness over a wide range of samples. We have shown that LCS is able to achieve scan times with an order of magnitude improvement over traditional raster scanning given the appropriate parameters, and even larger improvements when accounting for hardware limitations. While there is further work to be done to account for the increased variability and smoothness of edges on biological samples, its flexibility in the face of uncertainty regarding feature dimensions and orientation, as well as its retention of tracking capability at fast scanning speeds make it especially well suited for use in imaging dynamic biological systems. Due to its algorithmic nature, this technique can be implemented on existing instruments without making significant changes to the physical hardware.

## Figures and Tables

**Table 1:** Selection of results for calibration gratings, silicon step and λ-DNA. LCS times were experimentally determined; raster times were calculated based on tip speed and image dimensions of equivalent LCS scans as described in Section 2.1. A step size of *p* = 0.01 μm was used for all scans.

Sample	ω (Hz)	Radius (μm)	Tip Speed (μm/s)	LCS Time (s)	Raster Time (s), FOI-1	Raster Time (s), FOI-2	FOI-1	FOI-2
Square	20	0.10	12.57	102.14	1193.18	1193.18	11.68	11.68
Circle	20	0.10	12.57	56.31	342.15	342.15	6.08	6.08
Si (short)	100	0.30	188.50	3.83	102.57	12.00	26.78	3.13
Si (long)	100	0.30	188.50	11.50	746.84	48.73	64.95	4.24
DNA	60	0.16	60.32	11.88	302.04	41.39	25.42	3.48

## Abbreviations

AFM	Atomic Force Microscope
LCS	Local Circular Scan
FOI	Factor Of Improvement

## Appendix A. Sample Profile Estimator

The sample profile estimation technique used in this paper utilizes an observer of the dynamics of the cantilever, under an assumption that the AFM is operated at speeds close to the bandwidth of the *z*-piezo loop. A model of the cantilever can be expressed in discrete time form as:

(A1a) $$\begin{array}{c} x\left(k+1\right)=Fx\left(k\right)+Gu\left(k\right)+w_{1}\left(k\right)+w_{2}\left(k\right), \end{array}$$

(A1b) $$\begin{array}{c} y(k)=Hx(k)+v(k), \end{array}$$

where *F*, *G* and *H* are the state matrix, input matrix and output matrix, respectively, $w_{1}$ is a zero-mean, covariance $Q_{1}$, Gaussian white noise process that represents the input and thermal noise, v(k) is a zero-mean, covariance *R*, Gaussian white noise process that represents the measurement noise and $w_{2}\left(k\right)$ is the disturbance caused by the change in the sample profile, which we want to estimate.

By designing a steady-state Kalman observer that does not account for the $w_{2}$ term, information about this input disturbance can be extracted from the innovation signal. Through a series of algebraic manipulations detailed in [27], an estimate of the $w_{2}$ signal, and therefore of the sample profile, is given by:

(A2) $${\hat{w}}_{2}≃\sqrt{\left(1-f_{11}^{2}{\left(1-k_{11}\right)}^{2}\right)\left(\hat{\mathrm{var}}\left[\gamma \left(k\right)\right]-\bar{V}\right)}$$

where $f_{11}$ and $k_{11}$ are the first entries of the system and Kalman gain matrices, respectively, γ(k) is the innovation signal of the Kalman filter, $\hat{\mathrm{var}}\left[\gamma \left(k\right)\right]$ is an estimate of the variance of the innovation γ found by taking the mean square error over a finite window *M* and $\bar{V}=\frac{Q_{1}+R}{1-f_{11}^{2}{\left(1-k_{11}\right)}^{2}}$ is the expected steady state value of this variance at slow scan speeds. The signal is calibrated to units of nm by scanning a sample of known height; in this work, the Si steps’ grating was used. The entries in the state matrices can be determined through system identification of the specific cantilever used or, since the signal is calibrated based on a known sample, through the use of the manufacturer’s reported nominal values.
